# Effects of Mediterranean Diet on Endothelial Reactivity in Individuals with High Cardiometabolic Risk: A Randomized Controlled Parallel-Group Preliminary Trial

**DOI:** 10.3390/biomedicines12112595

**Published:** 2024-11-13

**Authors:** Roberta Lupoli, Ilenia Calcaterra, Pasquale Ambrosino, Rosalba Giacco, Marilena Vitale, Giuseppe Della Pepa, Angela Albarosa Rivellese, Gabriella Iannuzzo, Lutgarda Bozzetto, Matteo Di Minno

**Affiliations:** 1 Department of Molecular Medicine and Medical Biotechnology, Federico II University, 80131 Naples, Italy; roberta.lupoli@unina.it; 2 Department of Clinical Medicine and Surgery, Federico II University, 80131 Naples, Italy; ilenialorenza.calcaterra@unina.it (I.C.); marilena.vitale@unina.it (M.V.); giuseppe.dellapepa@unina.it (G.D.P.); rivelles@unina.it (A.A.R.); gabriella.iannuzzo@unina.it (G.I.); lutgarda.bozzetto@unina.it (L.B.); matteo.diminno@unina.it (M.D.M.); 3 Istituti Clinici Scientifici Maugeri IRCCS, Scientific Directorate of Telese Terme Institute, 82037 Telese Terme, Italy; 4 Institute of Food Sciences, National Research Council, 83100 Avellino, Italy; rosalba.giacco@isa.cnr

**Keywords:** mediterranean diet, endothelial function, inflammation, oxidative stress, cardiovascular risk, cardiovascular disability, chronic disease, exercise, lifestyle risk reduction, outcome

## Abstract

Background: Endothelial dysfunction is recognized as an early modification involved in the pathogenesis of vascular diseases. Evidence suggests that the Mediterranean Diet (MD) is associated with endothelial function improvement and, in turn, plays an important role in atherosclerosis development and progression. Objectives: To evaluate both acute and sustained effects of the MD on endothelial function in patients with high cardiometabolic risk. Methods: A total of 25 subjects were randomly assigned to either the MD group or the Control Diet (CD) group according to a single-blind, parallel-group study design. Endothelial function was evaluated through non-invasive flow-mediated dilation (FMD) measurements at baseline (T0) and after 8 weeks (Tw8) of the MD or CD intervention, under both 12 h fast condition (fasting) and 2 h post-meal resembling the assigned diet (2 h). Assessments were conducted by a blinded sonographer. Results: FMD at T0-fasting was similar between MD and CD groups (6.11% ± 0.67 vs. 7.90% ± 1.65; *p* = 0.266). A significant difference in FMD between MD and CD groups was observed at T0-2h (12.14% ± 1.93 vs. 4.01% ± 1.03; *p* = 0.004), T8w-fasting (9.76% ± 1.18 vs. 5.03% ± 0.89; *p* = 0.008), and T8w-2h (8.99% ± 1.22 vs. 3.86% ± 0.52; *p* = 0.003). Oral glucose insulin sensitivity (OGIS) at T0 correlated with FMD percent changes from T0-fasting to T0-2h (r = 0.414, *p* = 0.044). After adjusting for age, gender, and OGIS, MD was an independent predictor of percent changes in FMD from T0-fasting to T0-2h (β: −0.582, *p* = 0.003), from T0-fasting to T8w-fasting (β: −0.498, *p* = 0.013), and from T0-fasting to T8w-2h (β: −0.479, *p* = 0.018). Conclusions: Adherence to the MD may improve endothelial function in both the short- and medium-term among patients at high cardiometabolic risk.

## 1. Introduction

Cardiovascular (CV) disease is a leading cause of death worldwide. The World Health Organization estimates that approximately 20% of the population will be over 65 years old by the next decade and CV disease will be responsible for about 40% of deaths [1]. Atherosclerosis is a systemic and progressive condition, leading to coronary artery disease, cerebrovascular disease, and peripheral arterial disease [1]. Endothelial dysfunction has been recognized as the earliest alteration in the pathogenesis of atherosclerotic lesions and the subsequent onset of CV disease [2]. Indeed, the endothelium plays a key role in vascular health by secreting substances that regulate vasomotor tone and have antiatherogenic or antiproliferative functions [3], making it a potential therapeutic target for preventing CV disease and its associated disability [4]. The assessment of endothelial function in humans can be performed through several invasive and non-invasive clinical methods (e.g., peripheral artery tonometry, laser Doppler flowmetry), as well as by measuring various endothelial-derived mediators [5]. Among these, the assessment of flow-mediated dilation (FMD) of the brachial artery is an accurate, cost-effective, and non-invasive technique [6], used as a surrogate marker of subclinical atherosclerosis and an independent predictor of CV events [7]. Employing these methods, several data have suggested that endothelial function could be enhanced through lifestyle changes, such as specific dietary regimens [8,9] and exercise-based strategies, including rehabilitation [10,11].

Extensive evidence has shown that adherence to a Mediterranean dietary pattern (MD) is inversely associated with the risk of CV events and metabolic diseases [12,13]. In particular, literature findings suggests that the MD is associated with a lower risk of developing type 2 diabetes in both healthy subjects and in those with high CV risk [14,15]. Moreover, evidence from both observational cohort studies [16] and secondary prevention trials [17] has consistently found that adherence to the MD correlates with a reduced risk of CV events. This was later confirmed by the PREDIMED (Prevención con Dieta Mediterránea) randomized controlled trial, showing that the MD supplemented with extra-virgin olive oil or nuts is associated with a lower incidence of major CV events in high-risk subjects with no CV disease at enrolment [18]. The scientific literature has long investigated the mechanisms behind improvements in CV risk profiles, with clinical trials and meta-analyses suggesting that several components of the MD may enhance endothelial function [19,20,21,22,23]. However, while there is extensive evidence on the effects of medium-/long-term adherence to this dietetic regimen on endothelial function, few data exist about the acute effect of meals resembling MD.

Based on these premises, the aim of the present study was to evaluate: (1) the acute effects on endothelial function of an MD test meal resembling a typical Mediterranean lunch compared to a Western diet-type meal in obese/overweight subjects; and (2) the medium-term effect on endothelial function of a diet resembling a typical MD compared to a Western-type control diet (CD).

## 2. Subjects and Methods

### 2.1. Subject Recruitment

During a 12-month enrollment period, overweight/obese individuals of both genders, aged 20–65 year, attending the Federico II University Hospital Clinical Nutrition Center, Napoli, Italy, or working at the same institution, were assessed for eligibility for this study based on the following inclusion criteria: age between 20 and 65 years; a body mass index (BMI) of 25 to 35 kg/m^2^; habitual consumption of no more than 2 portions of wholegrain cereals and/or fiber-enriched foods per day limited to a maximum of 3 servings of fruit and vegetables daily); and a sedentary lifestyle.

Exclusion criteria were gastrointestinal disorders, pregnancy or breastfeeding, previous abdominal surgery, pharmacological treatments of any type at enrollment and in the 2 months prior to the study, habitual diet rich in fruit and vegetables, high level of physical activity, consumption of wine or alcohol equivalent beverage greater than 3 glasses of wine per day, concurrent participation in other studies, use of functional foods and/or food supplements of any kind.

This study was registered on ClinicalTrials.gov (Number: NCT03071718), was conducted according to the recommendations of the Declaration of Helsinki, and was approved by the Ethics Committee of Federico II University of Naples (Prot. N. 108/16). Signed, written informed consent was obtained from each participant. Whenever applicable, this study was conducted in accordance with the CONsolidated Standards Of Reporting Trials (CONSORT) guidelines [24].

### 2.2. Study Design and Protocol

**Figure 1:**
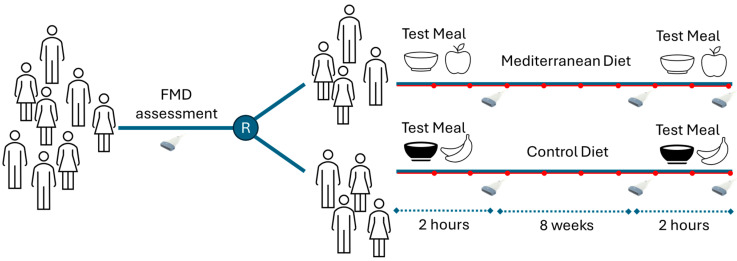
Study flow diagram. FMD: flow-mediated dilation. R: randomization.

As graphically represented in Figure 1, we designed a randomized controlled parallel-group trial with an 8-week nutritional intervention phase. Study participants, after a 2-week run-in period aimed at stabilizing their usual diet, were randomized to either MD regimen or CD regimen. Randomization was performed according to age, sex, and BMI of the subjects, using computer-generated random numbers.

The subjects assigned to the MD underwent an individual dietary intervention specifically designed to respect the features of the MD, maintaining the individual daily energy intake unchanged. Each volunteer received information on the type, weekly frequency, and quantity of foods to be consumed and those to be eliminated/reduced. Subjects assigned to CD continued to follow their usual diet. All subjects maintained their usual level of physical activity for the entire duration of the study. Compliance with nutritional interventions was assessed through 7-day food diaries that the volunteers filled in during the week before starting the intervention (baseline), the fourth (intermediate), and the eighth week of intervention (final).

In addition, a telephone interview was performed every 2 weeks in order to evaluate daily food consumption and physical activity during the previous 7 days and to check the progress of the protocol.

### 2.3. Experimental Diets and Test Meals

The MD group consumed an individualized diet that maintained the daily energy and macronutrient intake of each individual’s habitual diet and warranted a dietary pattern typical of the Mediterranean area. On the other hand, participants in the CD group maintained their habitual diet. The diets of the participants in the MD group were formulated to increase their individual intake of dietary fiber, plant vs. animal proteins, and monounsaturated and polyunsaturated fats vs. saturated fats. MD was designed to improve daily intake of fruit and vegetables (at least 5 portions, ~500 g/day) and nuts (30 g/day). Refined cereal products were replaced with wholegrain products (at least 2 portions, ~200 g/day between wholegrain pasta, bread, and breakfast cereal); meat and derived meat products with legumes and fish (at least 2 portions, ~300 g/week of fish and 3 portions, ~300 g/week of legumes); butter and any other fat source with extra-virgin olive oil. Participants were encouraged to consume meat, dairy products, and eggs only once a week. In contrast, subjects in the CD group were instructed to keep their habitual diet unchanged during the intervention and did not consume extra virgin olive oil. To improve adherence to the MD and CD, the main food products in both diets were supplied to participants in amounts sufficient to cover their household consumption for the whole study period. The MD and CD test meal compositions are reported in Table 1. Test meals were arranged in the metabolic kitchen by a dietitian with standardized amounts of foods in order to make the two meals similar in energy and macronutrient composition, but different in animal and vegetable protein sources, saturated fat, total fiber content, and glycemic index and load, thus reflecting the characteristics of either MD or CD. Food intake for eligibility screening and follow-up assessments was evaluated by qualified dietitians, using standardized food portions to assess energy intake.

### 2.4. Laboratory Assessment

Blood samples were drawn after a 12 h overnight fast and for 4 h during the test meal (every 30 min for the first 2 h and every 60 min for the following 2 h) for the measurement of blood glucose, insulin, and c-peptide. The concentration of total cholesterol, low-density lipoprotein cholesterol (LDL), high-density lipoprotein cholesterol (HDL), and triglycerides was also evaluated on the fasting sample.

### 2.5. Brachial Artery FMD

FMD was evaluated in each patient: (1) at baseline, before randomization after a 12 h fast (T0-fasting); (2) 2 h after an MD or CD meal test (T0-2h); (3) after 8 weeks of continuous adherence to the MD or CD in a 12 h fasting condition (Tw8-fasting); and (4) 2 h after an MD and CD meal test (Tw8-2h). The assessment of FMD was performed by the same operator in blinded conditions regarding the diet regimen. Patients were asked to abstain from alcohol, tobacco, and caffeine on the day of FMD assessment. All study procedures were performed in a temperature-controlled room (23 °C) after ≥10 min of rest in the supine position (a small head pillow was allowed).

FMD was measured by ultrasound imaging, as described in the guidelines of the International Brachial Artery Reactivity Task Force [6].

In each patient, we evaluated FMD of the brachial artery according to a standardized ultrasound protocol using an automatic edge detection software (Cardiovascular Suite^®^ Version 3, FMD studio, QUIPU Srl, Pisa, Italy). The examination consisted of measuring brachial artery diameter (BAD) at rest and after reactive hyperemia induced by ischemia of the forearm. The measurement was made on a B-mode section of the artery, which was imaged above the antecubital fossa in the longitudinal plane by using a linear ultrasound vascular transducer with a frequency of 10 MHz (Esaote^®^, MyLab 25 Gold, Pisa, Italy). Baseline BAD and the flow velocity were recorded for 60 s. The blood pressure cuff was placed on the forearm 4–5 cm behind the elbow joint line and inflated up to 70 mmHg above the systolic blood pressure to induce a transitory ischemia for 300 s. After cuff release, FMD was calculated as (Max diameter post-ischemic − Basal diameter)/Basal diameter × 100 and expressed as percent increase in BAD compared with the baseline value. The duration of the overall exam was about 10–15 min. The reproducibility of this scanning protocol was evaluated on a representative sample of 5 subjects randomly selected from the study population within 1 week from the first examination.

### 2.6. Statistical Analysis

Statistical analysis was performed with the IBM SPSS 29 system (SPSS Inc., Chicago, IL, USA). Continuous data were expressed as mean ± standard deviation (SD). The *t*-test was performed to compare continuous variables for paired samples and for independent samples. In the case of values with a skewed non-Gaussian distribution, Mann–Whitney U test was used to compare medians. The χ^2^ test or Fisher’s exact test were used to compare categorical variables. Relationships between continuous variables and FMD changes after test-meals were examined using simple regressions with Pearson’s correlation coefficient (r) for normally distributed variables and Spearman’s correlation (ρ) for non-parametric variables. All results were expressed as two-tailed values, with *p* values < 0.05 being statistically significant. To evaluate potential sources of heterogeneity, a sensitivity analysis was performed by stratifying patients according to major clinical and demographic characteristics.

### 2.7. Sample Size

Regarding sample size, with a pre-defined increase in FMD from T0 values to Tw8 ≥ 50%, 16 subjects (8 per group) were needed to obtain 80% power and a 5% α error. Also considering a drop-out risk, at least 20 subjects have been enrolled in the present study.

## 3. Results

**Figure 2:**
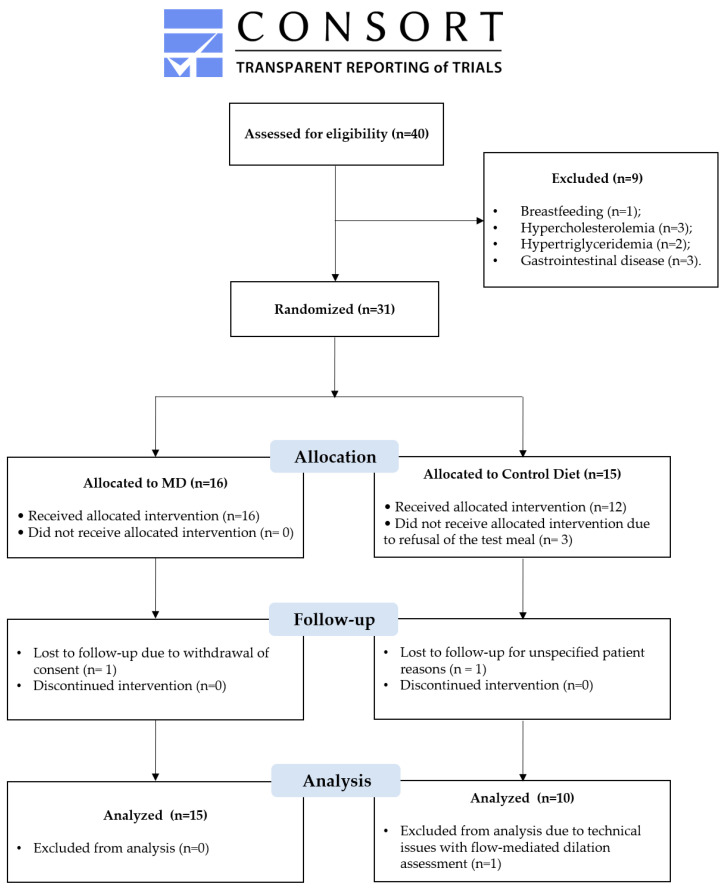
CONsolidated Standards Of Reporting Trials (CONSORT) flow chat of study participants.

Forty subjects were screened for inclusion in this study. Of these, nine did not meet the eligibility criteria. A total of 31 subjects were initially recruited, with two lost to follow-up (dropouts), three excluded for not participating in the test meal, and one excluded for not undergoing the FMD assessment. A total of 31 subjects was randomized, with 3 in the CD group excluded for not participating in the test meal, 2 lost to follow-up (one from each group), and 1 in the CD group excluded due to technical issues with the FMD assessment. As illustrated in Figure 2, 25 subjects were ultimately enrolled (mean age 42 ± 13 years; 51.7% female) and allocated randomized into the MD group (*n* = 15, 55.2%) and the CD group (*n* = 10, 44.8%).

At baseline, the MD group and CD group were comparable for major clinical and demographic characteristics (Table 2).

**Figure 3:**
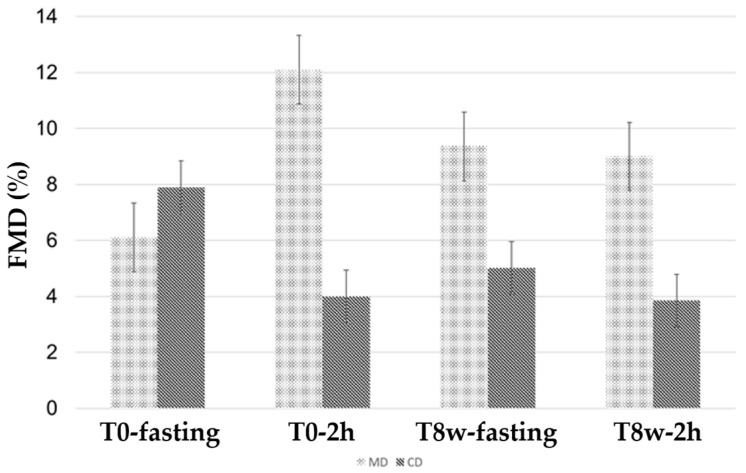
Flow-mediated dilation (FMD) in Mediterranean Diet (MD) and Control Diet (CD) groups at different time-points. 2h: 2 h after the test meal. 8w: 8 weeks after nutritional intervention.

Data on FMD assessment at T0-fasting, at T0-2h (acute effect), T8w-fasting (sustained effect), at T8w-2h (second meal test) are shown in Figure 3. FMD at T0-fasting was similar between MD and CD groups (6.11% ± 0.67 vs. 7.90% ± 1.65; *p* = 0.266). In contrast, we found a significant difference in FMD between the MD and CD group at T0-2h (12.14% ± 1.93 vs. 4.01% ± 1.03; *p* = 0.004), at T8w-fasting (9.76% ± 1.18 vs. 5.03% ± 0.89; *p* = 0.008), and at T8w-2h (8.99% ± 1.22 vs. 3.86% ± 0.52; *p* = 0.003).

**Figure 4:**
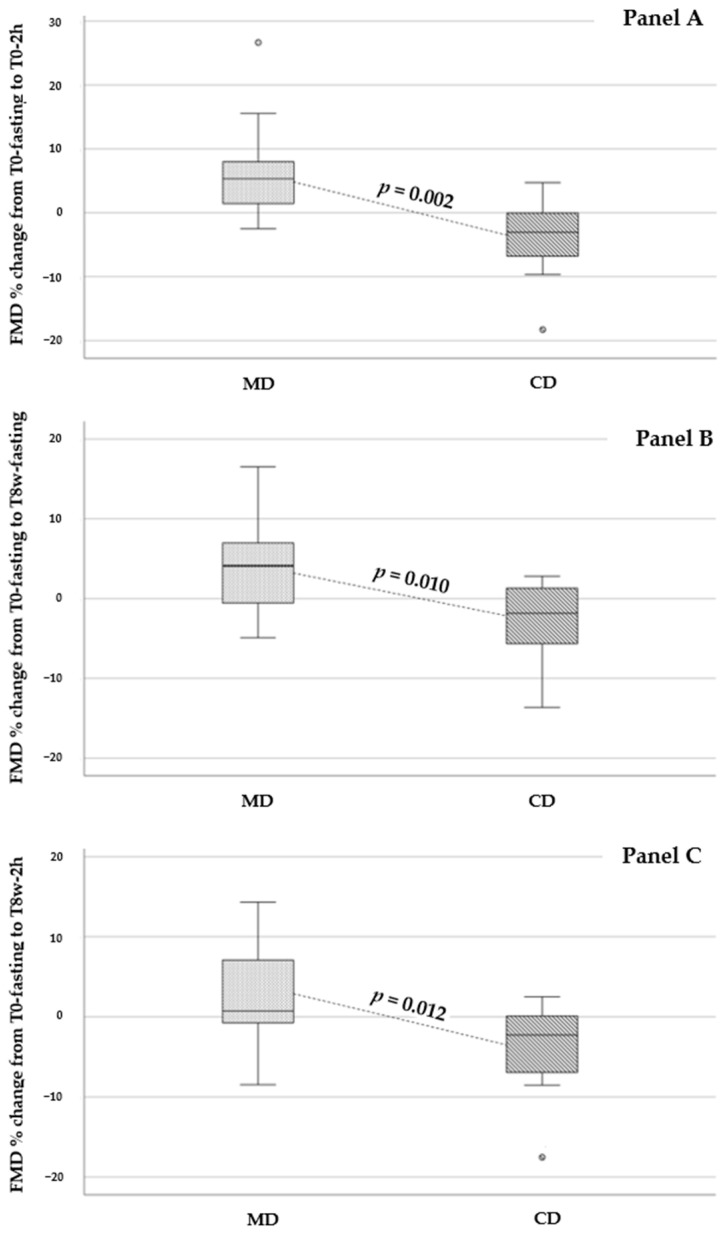
Percent changes in flow-mediated dilation (FMD) values from T0-fasting to T0-2h (Panel **A**), T8w-fasting (Panel **B**), and T8w-2h (Panel **C**) in Mediterranean Diet (MD) and Control Diet (CD) groups. 2h: 2 h after the test meal. 8w: 8 weeks after nutritional intervention.

Percent changes in FMD values from T0-fasting to T0-2h (acute effect), T8w-fasting (sustained effect), at T8w-2h (second meal test) were always significantly higher in MD group as compared to CD group (Figure 4).

T0-oral glucose sensitivity (OGIS) correlated with FMD percent changes from T0-fasting to T0-2h (r = 0.414, *p* = 0.044). T8w-OGIS correlated with FMD percent changes from T0-fasting to T8w-fasting (r = 0.469, *p* = 0.021) and showed a trend towards significant correlation with FMD percent changes from T0-fasting to T8w-2h (r = 0.378, *p* = 0.068). After adjusting for age, gender, and OGIS, MD was an independent predictor of percent changes in FMD from T0-fasting to T0-2h (β: −0.582, *p* = 0.003), from T0-fasting to T8w-fasting (β: −0.498, *p* = 0.013) and from T0-fasting to T8w-2h (β: −0.479, *p* = 0.018).

## 4. Discussion

The present study demonstrated that adherence to MD is associated with a significant improvement of endothelial function in overweight/obese subjects.

These results align with the previously published literature data, including meta-analytic evidence, showing that the MD exerts beneficial effects on both functional (FMD) and structural (IMT) measures of sub-clinical atherosclerosis, with a stronger effect for the functional ones [9,23]. Clinical trials investigating the effects of single components of the MD, such as nuts [20,25], fish [21,26], and olive oil [22,27], have shown an improvement in endothelial function. In this regard, data from the CORDIOPREV (CORonary Diet Intervention with Olive oil and cardiovascular PREVention) study suggest that the MD better modulates endothelial function compared with a low-fat diet and is also associated with a better vascular homeostasis in patients with coronary artery disease [28]. These results are confirmed by a recent meta-analysis showing that the MD improves endothelial function in adults, suggesting that its protective effects are evident at early stages of the atherosclerotic process with important implications for the early prevention of CV disease and its outcomes [23]. Nevertheless, although there is substantial evidence concerning the impact of medium- to long-term adherence to this dietary regimen on endothelial function, there is a scarcity of data regarding the acute effects of meals that resemble the MD. Meals in general [26,29], particularly those rich in saturated fats [30,31], cause postprandial alteration of metabolic variables and endothelial function, which is more frequently observed in subjects with CV risk factors [32] and could significantly impact CV risk. Therefore, it is important to understand whether the improvement of endothelial function associated with the MD could be driven by an acute effect in the postprandial phase.

In our study, we showed an improvement in FMD after an acute exposure during a meal test resembling a complete MD lunch (acute effect), as well as after 8-week adherence to MD (sustained effect). To the best of our knowledge, this is the first study evaluating both acute and sustained effects of the MD on endothelial function. In addition, its beneficial effects on FMD were entirely confirmed during a second meal test. The literature data on the acute (postprandial) effect of the MD on FMD are scanty and mostly focused on specific components of MD [33,34,35]. A study by Karatzi et al. showed that acute combined consumption of red wine and green olive oil, two essential components of the MD, led to an improvement in the postprandial endothelial function in 15 healthy subjects [34], whereas a high-saturated fatty acid meal induced postprandial endothelial dysfunction in 28 healthy males without overt CV risk factors [33].

In our study, whereas baseline BAD and FMD were similar in the two treatment groups, we observed significantly higher FMD percent changes in MD as compared to CD at all time-points. These results suggest an acute and beneficial effect of MD, consistently maintained as a sustained effect.

From a pathophysiological point of view, different underlying mechanisms through which MD components can improve endothelial function both in acute and chronic settings can be involved. In detail, it is well known that most MD components are able to increase the bioavailability of nitric oxide (NO), thus improving vascular reactivity [36]. In the short term, the MD can induce antioxidant effects resulting in a reduced superoxide scavenging of NO [36,37] and provide NO precursors inorganic nitrate (green leafy vegetables) and L-arginine (from nuts, grains, legumes, and fish) [37]. In the medium- and long-term, it modulates the up regulation of endothelial NO synthase, reduces the oxidation of LDL, and exerts anti-inflammatory effects [38,39], with outcomes that are similar to [40,41] and possibly synergistic with those of physical training and exercise-based interventions (e.g., rehabilitation) [42,43]. Among anti-inflammatory effects, an MD regimen is able to modulate inflammatory molecules, including interleukin-6 (IL-6), C-reactive protein (CRP), tumor necrosis factor-α (TNF-α), vascular cell adhesion protein-1 (VCAM-1), and soluble intercellular adhesion molecule-1 (sICAM-1) [44,45]. These effects seem to be mediated by down-regulation of the Nuclear Factor kappa-light-chain-enhancer of activated B cells (NF-kB) pathway [46] and altered methylation of inflammation-related genes [45]. The improvement in FMD could also be influenced by insulin sensitivity, a link between insulin resistance and endothelial dysfunction well known [47,48]. Interestingly, we documented a significant correlation between OGIS and FMD percent changes, both at baseline and at the 8-week assessment. A further key role can be attributed to visceral fat secretion of pro-inflammatory adipokines and cytokines, resulting in higher oxidative stress and lower NO bioavailability [49]. Moreover, insulin stimulates Mitogen-Activated Protein Kinase (MAPK)-dependent endothelin-1 (ET-1) synthesis and secretion, as well as Phosphoinositide 3-Kinase (PI3K)-dependent NO production from the vascular endothelium to maintain normal vascular tone [50]. Insulin resistance leads to endothelial derangements secondary to an overdriven MAPK-dependent signaling (increased ET-1 synthesis and secretion), together with impaired PI3K-dependent signaling (NO production) in the vascular endothelium [50]. Although the improvement in insulin sensitivity may have contributed to the improvement in endothelial function, it is important to highlight that, after adjusting for age, gender, and OGIS, MD was an independent predictor of both acute and sustained changes in FMD, suggesting a direct effect of MD.

From a clinical point of view, the impact of MD on CV event reduction could be significant, considering that a 1% reduction in FMD is associated with a 9–13% increase in CV risk [51,52]. Thus, the changes observed in FMD values in our sample during the MD intervention could potentially translate into a 30–35% reduction in the risk of CV events. Naturally, this is only a possible interpretation of our results, which, albeit preliminary and still inconclusive, can offer insights for future research with a rigorous design to evaluate whether the impact of the MD on endothelial function could play a role in the primary and secondary prevention of CV disease and Dity.

One of the major strengths of our study is represented by the blinded FMD assessment. The operator was not aware of the dietary treatment assigned to each participant, limiting the sources of bias and making the measurements more reliable. Furthermore, to reduce operator bias and optimize repeatability of the results, we used software that enables the automated assessment of vessel diameter and flow velocity. Another strength point is the study design conducted as a randomized controlled trial to maximally reduce the impact of bias on our results.

Some limitations of our study should also be discussed. A factor potentially affecting the comparison of FMD in independent patient groups is a difference in resting BAD [53]. For this reason, in the present study we measured resting BAD, which resulted similar between the MD and CD groups, thus ruling out such a possibility. Another potential limitation is the small sample size and the fact that the randomization ratio is not fully balanced. Although the sample size calculation was performed and adhered to in order to estimate the target enrollment number, such calculations for small-sized studies can be somewhat artificial and should be interpreted with caution [54]. On the other hand, the observation that our results have a strong statistical significance makes us confident about their reproducibility and generalizability. Moreover, we enrolled a specific population represented by overweight/obese subjects without concomitant metabolic alterations, potentially limiting the generalizability of our findings to other clinical settings with overlapping metabolic diseases. However, it is important to highlight that this group of individuals constitutes a significant portion of the general population, making our findings relevant for CV primary and secondary prevention. Another limitation to consider is that, although the ultrasound operator performing the FMD assessments was blinded, it was inevitable that the experienced dietitians overseeing the patients could not operate under the same conditions. However, as per study protocol, both the sonographer and dietitians were strictly instructed not to discuss the diet type throughout the study, which partially mitigates this limitation. Finally, it should be noted that our results are based on a clinical method for assessing endothelial function, and although its reproducibility and reliability have been widely demonstrated [55], our study did not include any pro-inflammatory markers or acute reactant cytokines to corroborate our findings.

## 5. Conclusions

In conclusion, our study demonstrates that the consumption of the MD improves endothelial function in overweight/obese subjects. This effect is clear even after a single meal, thus suggesting an acute beneficial effect that is consistently sustained over time. Our data strongly support dietary intervention as a key element of CV prevention.

## Figures and Tables

**Table 1:** Composition of test meals for Mediterranean and control diets.

Mediterranean Diet Food Component	Weight	Control Diet Food Component	Weight
Dried Beans	*80* g	Rice	*80* g
Cod	*50* g	Parmesan	*30* g
Arugula	*100* g	Bresaola	*60* g
Wholegrain Bread	*120* g	White Bread	*30* g
Extra-virgin Olive Oil	*29* g	Olive Oil	*21* g
Orange	*150* g	Banana	*160* g

**Table 2:** Clinical and demographic characteristic of study population.

	Mediterranean Diet *n* = 15	Control Diet *n* = 10	*p* Value
Age (years)	41 ± 12	44 ± 14	0.616
Weight (kg)	82.7 ± 13.6	80.8 ± 13.3	0.752
BMI (kg/m^2^)	29.2 ± 2.4	29.3 ± 4.3	0.948
Waist Circumference (cm)	97.1 ± 10.2	95.5 ± 6.9	0705
Hip Circumference (cm)	109.6 ± 7.3	109.5 ± 8.5	0.994
Cholesterol (mg/dL)	190 ± 41	196 ± 35	0.707
Triglycerides (mg/dL)	98 ± 42	101 ± 37	0.886
HDL-C (mg/dL)	49 ± 10	48 ± 11	0.902
LDL-C (mg/dL)	122 ± 37	128 ± 33	0.678
Glycemia (mg/dL)	95 ± 8	102 ± 9	0.068
SBP (mm/Hg)	110 ± 15	119 ± 15	0.188
DBP (mm/Hg)	71 ± 14	76 ± 7	0.325

Abbreviations: BMI: Body Mass Index; HDL-C: High Density Lipoprotein Cholesterol; LDL-C: Low Density Lipoprotein Cholesterol; SBP: Systolic Blood Pressure; DBP: Diastolic Blood Pressure. Baseline basal brachial artery diameter (BAD) of the overall population was 3.81 mm ± 0.89, without any difference between MD and CD (3.62 mm ± 0.88 vs. 4.09 mm ± 0.87, *p* = 0.215).

## Data Availability

The data supporting the findings of this study are available from the corresponding authors upon reasonable request due to privacy/ethical restrictions.
